# NeuroNet-AD: A Multimodal Deep Learning Framework for Multiclass Alzheimer’s Disease Diagnosis

**DOI:** 10.3390/bioengineering12101107

**Published:** 2025-10-15

**Authors:** Saeka Rahman, Md Motiur Rahman, Smriti Bhatt, Raji Sundararajan, Miad Faezipour

**Affiliations:** 1 School of Engineering Technology, Electrical and Computer Engineering Technology, Purdue University, West Lafayette, IN 47907, USA; rahma122@purdue.edu (S.R.); rahma112@purdue.edu (M.M.R.); 2 School of Applied and Creative Computing, Purdue University, West Lafayette, IN 47907, USA; bhatt32@purdue.edu

**Keywords:** Alzheimer’s disease diagnosis, multimodal fusion, deep learning, CBAM, MGCA

## Abstract

Alzheimer’s disease (AD) is the most prevalent form of dementia. This disease significantly impacts cognitive functions and daily activities. Early and accurate diagnosis of AD, including the preliminary stage of mild cognitive impairment (MCI), is critical for effective patient care and treatment development. Although advancements in deep learning (DL) and machine learning (ML) models improve diagnostic precision, the lack of large datasets limits further enhancements, necessitating the use of complementary data. Existing convolutional neural networks (CNNs) effectively process visual features but struggle to fuse multimodal data effectively for AD diagnosis. To address these challenges, we propose NeuroNet-AD, a novel multimodal CNN framework designed to enhance AD classifcation accuracy. NeuroNet-AD integrates Magnetic Resonance Imaging (MRI) images with clinical text-based metadata, including psychological test scores, demographic information, and genetic biomarkers. In NeuroNet-AD, we incorporate Convolutional Block Attention Modules (CBAMs) within the ResNet-18 backbone, enabling the model to focus on the most informative spatial and channel-wise features. We introduce an attention computation and multimodal fusion module, named Meta Guided Cross Attention (MGCA), which facilitates effective cross-modal alignment between images and meta-features through a multi-head attention mechanism. Additionally, we employ an ensemble-based feature selection strategy to identify the most discriminative features from the textual data, improving model generalization and performance. We evaluate NeuroNet-AD on the Alzheimer’s Disease Neuroimaging Initiative (ADNI1) dataset using subject-level 5-fold cross-validation and a held-out test set to ensure robustness. NeuroNet-AD achieved 98.68% accuracy in multiclass classification of normal control (NC), MCI, and AD and 99.13% accuracy in the binary setting (NC vs. AD) on the ADNI dataset, outperforming state-of-the-art models. External validation on the OASIS-3 dataset further confirmed the model’s generalization ability, achieving 94.10% accuracy in the multiclass setting and 98.67% accuracy in the binary setting, despite variations in demographics and acquisition protocols. Further extensive evaluation studies demonstrate the effectiveness of each component of NeuroNet-AD in improving the performance.

## 1. Introduction

Advancements in healthcare have increased the average global life expectancy. It is projected that about 90% of the countries will be considered aged societies, and more than 50% will be ultra-aged by 2100 [1]. This demographic change has a profound impact on the care of old people, particularly in relation to dementia, a neurodegenerative disease often developed in the aging population. Alzheimer’s disease (AD) is the most common type of dementia, which includes up to 80% of total dementia [2]. Characterized by the presence of amyloid-β (Aβ) plaques and tau-containing neurofibrillary tangles (NFTs), AD significantly impacts the quality of life with a decline in cognitive ability that interferes with daily activities [3]. The progression of AD includes different stages, from preclinical and very mild cognitive impairment (MCI) to mild and severe stages [4].

AD diagnosis involves clinical examinations and interviews with patients and their family members [5]. Clinicians often request additional pathological tests to identify patients more accurately [6]. Imaging techniques such as Positron Emission Tomography (PET) and Magnetic Resonance Imaging (MRI), which are widely accessible and noninvasive imaging modalities, are commonly utilized for the diagnosis and treatment of AD [7,8,9]. There are several commonly used screening tools to measure AD, including the Clinical Dementia Rating (CDR) and the Mini-Mental State Examination (MMSE) [10,11]. However, early diagnosis of AD is challenging as studies show that the misdiagnosis rates of probable AD are above 16% [12]. These limitations underscore the need for advanced computational methods such as machine learning (ML) and deep learning (DL) to improve diagnosis and management.

Numerous researchers have applied ML algorithms including support vector machines (SVMs), Random Forests (RFs), logistic regression (LR), naive Bayes (NB), and multilayer perceptrons (MLPs) for AD diagnosis [4,13,14,15,16,17,18]. The performance of these algorithms depends on manual feature extraction techniques that require domain expertise and are labor intensive [19,20]. Deep learning, particularly convolutional neural networks (CNNs), has addressed these limitations by automating feature extraction from MRI and PET scans, improving diagnostic accuracy [21,22,23,24]. CNNs, however, struggle with capturing temporal changes for AD progression [25]. Recurrent Neural Networks (RNNs), including Long Short-Term Memory (LSTM) and Gated Recurrent Unit (GRU), overcome the problem of capturing long-term dependencies in time-series data such as Electroencephalogram (EEG) signals and clinical assessments [26,27,28,29,30,31]. Recent advances have made natural language processing (NLP) an essential tool for analyzing language and speech patterns that are indicators of cognitive decline [32,33]. Models such as Bidirectional Encoder Representations from Transformers (BERT) and the Generative Pre-trained Transformer (GPT) analyze changes in fluency and complexity to detect neurological impairments [32,33,34,35,36]. On the other hand, vision transformers (ViTs) have advanced the use of medical imaging for AD diagnosis by capturing local and global dependencies through self-attention mechanisms [25,37,38,39,40,41], which requires significantly more computation than CNN models.

Along with imaging data, healthcare providers use patient records, neurological tests, and genetic history to diagnose AD [42]. This approach leads to the collection of various data such as imaging, biological markers, and clinical evaluation in patients [43]. Although Computed Tomography (CT), MRI, and PET images provide crucial insights into the disease, incorporating patient records, neurological tests, and genetic history is equally important for diagnosing Alzheimer’s disease. Given the challenges in acquiring more images from a larger patient population, it is vital to leverage the available data to enhance diagnostic precision. While CNN models excel with imaging data, their current frameworks fall short in effectively utilizing multimodal information for improved performance [42]. Moreover, most of the existing multimodal models struggle as they compute attentions separately from each modality. These challenges limit the clinical applicability and generalizability of such models in AD diagnosis. In addition, most studies focus on diagnosing AD from normal control (NC) [44]. However, MCI is a preliminary stage that is considered a transition state from NC to AD dementia [45]. Since there is currently no treatment for AD, accurately diagnosing the disease in its early stage is important to provide patient care and develop future treatments. We propose a novel multimodal deep learning framework that effectively integrates imaging data (MRI), textual clinical data, demographic information, genetic biomarkers, and cognitive test scores to diagnose AD, MCI, and NC. This approach aims to improve diagnostic accuracy and interpretability by utilizing an improved feature aggregation strategy that captures cross-modal interactions.

The main contributions of our work are as follows:

- We propose NeuroNet-AD, a novel multimodal deep learning framework that integrates MRI images with clinical text-based metadata for improved multiclass AD classification with the utilization of the Convolutional Block Attention Module (CBAM) and the Meta-Guided Cross-Attention (MGCA) mechanism. These attention computations allow the model to focus on important features and allow for better cross-modality data fusion.
- We employ an ensemble-based feature selection strategy combining Random Forest, eXtreme Gradient Boosting (XGBoost), Light Gradient Boosting Machine (LightGBM), ExtraTrees, and AdaBoost, following majority voting to identify the top-most discriminative features from the clinical text data that contribute the most to the performance.
- We conduct comprehensive experiments using the Alzheimer’s Disease Neuroimaging Initiative (ADNI) dataset to validate the performance of NeuroNet-AD along with the other state-of-the-art (SOTA) models. We also quantify the impact of each component of NeuroNet-AD on the performance to justify its configuration.

The remainder of this paper is organized as follows: Related works are discussed in Section 2. Section 3 illustrates the methods proposed and employed in this work. Section 4 presents the implementation of the model and the description of the dataset. The results obtained are reported in Section 5, and Section 6 concludes the paper.

## 2. Related Works

The multimodal approach of integrating neuroimaging with clinical textual data offers a more comprehensive understanding of the complex and diverse nature of AD [46,47]. This approach allows for early detection and accurate monitoring of the progression of the disease [48,49,50].

Golovanevsky et al. (2022) [42] presented an attention-based multimodal Alzheimer’s disease diagnosis (MADDi) model to detect AD and MCI through the integration of imaging, genetic, and clinical data and achieved an accuracy of 96.88% on the ADNI dataset. In another study, Wisely et al. (2022) [51] developed a convolutional neural network (CNN) to detect AD from NC using multimodal retinal images and patient data. The retinal image dataset consisted of 284 eye images from 159 subjects. The model achieved the highest performance with an Area Under the Curve (AUC) of 0.809 using only the image dataset, while the full multimodal model incorporating all imaging data, quantitative metrics, and patient data achieved an improved AUC of 0.836. Altaf et al. (2018) [52] presented a model integrating feature descriptors such as the Gray Level Co-occurrence Matrix (GLCM), Scale-Invariant Feature Transform (SIFT), Local Binary Pattern (LBP), and Histogram of Oriented Gradients (HOG) to extract information from MRI images. In addition, the study combined clinical data with image-based features to form a comprehensive hybrid feature vector. The proposed model was validated on the ADNI dataset, achieving an accuracy of 98.4% for binary classification (AD vs. NC) and 79.8% for multiclass classification (AD, NC, and MCI).

Recent advances in vision language pre-training (VLP) have shown promising applications in medical diagnosis, particularly by integrating multimodal data such as images (X-rays and MRIs) and text such as doctors’ notes, electronic health records (EHRs), or histories [53]. Some recent works in AD diagnosis have utilized the VLP model using large-scale medical image and text data to improve interpretability and classification accuracy. Chen and Hong (2024) [54] developed Medical Bootstrapping Language Image Pre-training (MedBLIP), a lightweight computer-aided diagnosis (CAD) system that uses 3D medical images and text data using a query-based mechanism. The model detected NC, MCI, and AD using frozen pre-trained encoders and parameter-efficient fine-tuning techniques, achieving an accuracy of 78.7% on the ADNI dataset, 83.3% on the National Alzheimer’s Coordinating Center (NACC) dataset, and 85.3% on the Open Access Series of Imaging Studies (OASIS) dataset. In zero-shot evaluation, MedBLIP demonstrated impressive performance with 80.8% accuracy on the Australian Imaging, Biomarkers & Lifestyle (AIBL) dataset and 71.0% on the Minimal Interval Resonance Imaging in Alzheimer’s Disease (MIRIAD) dataset.

Lee et al. (2025) proposed a graph neural network approach utilizing a vision–language model (VLM) to map image–text relationships for dementia detection [55]. The method, employing Bootstrapping Language Image Pre-training (BLIP) and graph convolutional networks (GCNs), achieved an accuracy of 88.73% to detect NC and AD on the Alzheimer’s Dementia Recognition through Spontaneous Speech (ADReSSo Challenge) dataset. In another study, Feng et al. (2023) [56] introduced a framework employing large language models (LLMs) with convolutional neural networks (CNNs) and transformers to fuse image and non-image data. This approach used cross-attention mechanisms and prompt tuning to align modalities. The experiments on the ADNI dataset achieved an accuracy of 96.36% for the AD vs. NC classification and 94.71% for the early MCI (EMCI) vs. late MCI (LMCI) classification.

Finally, Chiumento et al. (2024) [57] introduced a framework using synthetic diagnostic reports generated from structured clinical and MRI data to train the Biomedical Contrastive Language–Image Pre-training (BiomedCLIP) and T5 (Text-to-Text Transfer Transformer) models. Their model’s performance was evaluated using the Bilingual Evaluation Understudy (BLEU-4) (0.1827), Recall-Oriented Understudy for Gisting Evaluation on Longest common subsequence (ROUGE-L) (0.3719), and Metric for Evaluation of Translation with Explicit Ordering (METEOR) (0.4163) scores on the OASIS-4 dataset for NC vs. MCI vs. AD classification.

While existing multimodal approaches enhance AD diagnosis, many models lack an effective feature aggregation strategy that fully captures cross-modal interactions, limiting their interpretability and robustness. To address this, we propose NeuroNet-AD, a novel multimodal framework that integrates MRI images with clinical text metadata. NeuroNet-AD employs the Convolutional Block Attention Module (CBAM) and Meta-Guided Cross-Attention (MGCA) to enhance feature fusion, alongside an ensemble-based feature selection strategy for improved discriminative power. Our comprehensive experiments on the ADNI dataset validate its effectiveness against SOTA models.

## 3. Methods

### 3.1. Problem Statement

We denote $\left(X_{i},Y_{i},i=1,\ldots ,N\right)$ as the image *X* and class label *Y* spaces with distribution D. In supervised deep learning, the model is trained to learn the data distribution for classifying Alzheimer’s disease using $F_{\theta }\left(X\right)\to \hat{Y}$, aiming for $\hat{Y}$ (the predictions) to be as similar as possible to *Y* (the labels). Typically, this type of learning has primarily been conducted to enhance classification performance, though other complementary information may further improve the performance. Age, weight, and various cognitive test scores, such as the Mini-Mental State Examination (MMSE), Functional Activities Questionnaire (FAQ), Global Clinical Dementia Rating (Global CDR), and Neuropsychiatric Inventory Questionnaire (NPIQ) scores, contain valuable complementary information; thus, proper fusion of this metadata with visual features is crucial for enhancing the performance. In this study, we propose a novel model $F_{\theta }\left(X,X_{t}\right)\to \hat{Y}$ to effectively fuse image *X* and metadata (textual data) $X_{t}$, predicting $\hat{Y}$ to be closer to *Y*.

### 3.2. Method Overview

**Figure 1:**
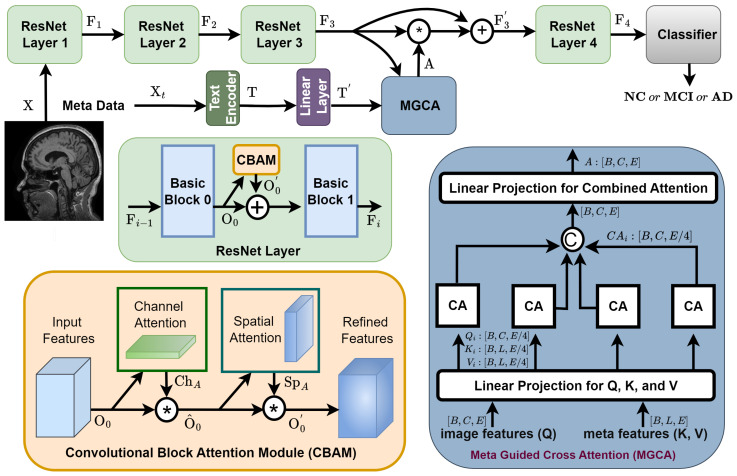
Pipeline of our NeuroNet-AD model. The framework consists of three main components: (1) ResNet-based image feature extractor, enhanced with CBAM to refine spatial and channel-wise information; (2) text encoder, which processes meta-information (e.g., textual descriptions and structured metadata) using a BERT-based transformer model; (3) MGCA module, which fuses image and text features using a multi-head cross-attention mechanism.

This study presents a novel NeuroNet-AD model that utilizes image and meta-features using Meta-Guided Cross-Attention (MGCA) for better fusion of both image and text modalities to improve the diagnosis of AD stages. NeuroNet-AD, as presented in Figure 1, has four main modules, including ResNet-18 with Convolutional Block Attention Modules (CBAMs), a Text Encoder, Meta-Guided Cross-Attention (MGCA), and a final classification layer. The model utilizes a ResNet-18 backbone for image feature extraction, enhanced by CBAM to refine important spatial and channel-wise features. We chose ResNet-18 because its moderate depth balances feature capacity with overfitting risk on limited data. A pre-trained BERT model is used as the text encoder to generate language embeddings for incorporating text information. These embeddings are fused with image features through the MGCA mechanism, facilitating better cross-modal feature alignment. Finally, a classifier processes the combined feature representation to produce the final result.

#### 3.2.1. ResNet Layers with CBAM

Proper attention computation and utilization are essential to improve the performance of a deep learning model. Attention utilization during feature extraction helps the model to focus more on the important regions and to converge faster. Hence, in NeuroNet-AD, we introduce CBAM attention computation in each ResNet layer to enhance its feature extraction. Each ResNet layer has two basic blocks numbered 0 and 1, where each basic block consists of several sequential operations, including convolution, batch normalization, and the Rectified Linear Unit (ReLU). We incorporate the CBAM between the two ResNet blocks (0 and 1) as shown in Figure 1, which we implement through Equation (1).

(1) $$O_{0}=BasicBlock_{0}\left(F_{i-1}\right);\;\;\;\;\;O_{0}^{\prime }=CBAM\left(O_{0}\right);\;\;\;\;\;F_{i}=BasicBlock_{1}\left(O_{0}+O_{0}^{\prime }\right)$$

Here, $F_{i-1}$ denotes the input feature map to the $i^{\mathrm{th}}$ ResNet layer, $O_{0}$ is the output of the first block (basic block 0) within ResNet layer *i*, $O_{0}^{\prime }$ is the CBAM-refined version of $O_{0}$, and $F_{i}$ represents the output feature map of ResNet layer *i*. CBAM enhances feature maps by applying channel and spatial attention sequentially. First, channel attention $\left(Ch_{A}\right)$ computes importance across channels using average and max pooling, as shown in Equation (2). The computed channel attention $\left(Ch_{A}\right)$ is multiplied by the input features $\left(O_{0}\right)$, resulting in improved input features $\left({\hat{O}}_{0}\right)$, which we pass through to compute spatial attention. The spatial attention $\left(Sp_{A}\right)$ shown in Equation (3) refines spatial features using average and max pooling along the channel axis.

(2) $$Ch_{A}=\sigma \left(\mathrm{MLP}\left(\mathrm{AvgPool}\left(O_{0}\right)\right)+\mathrm{MLP}\left(\mathrm{MaxPool}\left(O_{0}\right)\right)\right)$$

(3) $${\hat{O}}_{0}=O_{0}*Ch_{A};\;\;\;\;\;Sp_{A}=\sigma \left(\mathrm{Conv}\left(\left[\mathrm{AvgPool}\left({\hat{O}}_{0}\right),\mathrm{MaxPool}\left({\hat{O}}_{0}\right)\right]\right)\right)$$

where σ denotes the sigmoid function, MLP represents a multi-layer perceptron, and Conv is the convolution operation. Finally, the computed spatial attention is multiplied element-wise with the improved input features to obtain the refined feature $\left(O_{0}^{\prime }\right)$ of the CBAM module, shown in Equation (4).

(4) $$O_{0}^{\prime }=Sp_{A}*{\hat{O}}_{0}$$

We follow this technique to add CBAM in each layer to make the ResNet layers more efficient for feature extraction.

#### 3.2.2. Text Encoder

A text encoder processes structured and unstructured textual information (such as clinical metadata, diagnostic notes, or text-based descriptions) and transforms it into feature embeddings that can be fused with image features. We have used a pre-trained BERT (Bidirectional Encoder Representations from Transformers) model as our text encoder for the metadata. The tokenized textual input $X_{t}$ is fed to the BERT model to generate the contextual embedding T of the text input, illustrated in Equation (5).

(5) $$T=\mathrm{BERT}(X_{t})$$

We pass the generated embedding through a learnable linear layer to map the shape with the encoded visual features. The final hidden states from BERT are projected into a feature space compatible with the ResNet image features, shown in Equation (6).

(6) $$T^{\prime }=W_{T}T+b_{T}$$

where $W_{T}$ and $b_{T}$ are the learnable parameters.

#### 3.2.3. Meta-Guided Cross-Attention (MGCA)

The Meta-Guided Cross-Attention (MGCA) module is a multimodal feature fusion mechanism between visual and text features. This module utilizes a multi-head (four heads) cross-attention mechanism to enable effective alignment between image and textual representations, as shown in Figure 1. The embedding dimension was split into four equal segments to match the four attention heads in MGCA, improving computational efficiency and enabling each head to learn complementary cross-modal interactions. The MGCA module takes two primary inputs: (1) image features: $F_{3}\in R^{B\times C\times E}$ extracted from the third residual block and (2) text features: $T^{\prime }\in R^{B\times L\times E}$ obtained from the BERT-based text encoder, where *B* is the batch size, *C* is the number of feature channels, *L* is the sequence length of the text embeddings, and *E* is the embedding dimension of both image and text features. We maintain the same values of *C* and *L* as 256 to perform the computation. The input features are projected onto a lower-dimensional space before being applied to cross-attention to improve computational efficiency. Since we use four attention heads, we divide the embedding into four segments and perform the cross-attention (CA). The transformation is given by Equation (7).

(7) $$Q_{i}=W_{Q_{i}}F_{3},\;K_{i}=W_{K_{i}}T^{\prime },\;V_{i}=W_{V_{i}}T^{\prime }$$

where $Q_{i}\in R^{B\times C\times E/4}$ is the query matrix derived from image features; and $K_{i}\in R^{B\times L\times E/4}$ and $V_{i}\in R^{B\times L\times E/4}$ are the key and value matrices, respectively, derived from the text embeddings for attention head *i*. Here, $W_{Q_{i}},W_{K_{i}},$ and $W_{V_{i}}$ are learnable projection matrices that reduce the embedding size from *E* to E/4 for attention head *i*. Then, the MGCA module employs cross-attention (CA) to establish correspondences between the two modalities. For each attention head *i*, the attention mechanism is computed as presented in Equation (8).

(8) $${\mathrm{CA}}_{i}=\mathrm{Attention}\left(Q_{i},K_{i},V_{i}\right)=\mathrm{softmax}\left(\frac{Q_{i}K_{i}^{T}}{\sqrt{E/4}}\right)V_{i}$$

After computing the attention outputs from four heads, the outputs are concatenated and passed through a linear projection layer, which generates the final attention $A\in R^{\left(B\times C\times E\right)}$. The computed attention (A) is incorporated with the image features using the residual connection $\left(F_{3}^{\prime }=\left(A*F_{3}\right)+F_{3}\right)$ before going to the next residual block.

#### 3.2.4. Final Classifer

The final classification layer maps the features to the target classes. The output of the fourth residual block $\left(F_{4}\right)$ is flattened and then fed to a sequential fully connected (FC) layer for classification, as shown in Equation (9).

(9) $$\hat{Y}=Softmax\left(\mathrm{FC}\left(\mathrm{flatten}\left(F_{4}\right)\right)\right)$$

The model is trained using categorical cross-entropy loss shown in Equation (10).

(10) $$L=-\sum_{j=1}^{N}y_{j}\log\left({\hat{y}}_{j}\right)$$

where $y_{j}$ is the true one-hot label for class *j*, ${\hat{y}}_{j}$ is the predicted probability for class *j*, and *N* is the number of classes.

## 4. Experiments

### 4.1. Dataset

The dataset used for this research was collected from the Alzheimer’s Disease Neuroimaging Initiative (ADNI) study: a longitudinal, multi-center, observational dataset [58]. We used the ADNI1 version, which includes 3D MRI images and related metadata from 200 subjects. These subjects encompass Normal controls (NCs), individuals with mild cognitive impairment (MCI), and patients diagnosed with Alzheimer’s disease (AD). Along with imaging data, the dataset offers several clinically relevant metadata fields: Weight, Age, APOE-A1, APOE-A2 (Apolipoprotein E alleles associated with AD risk), MMSE (Mini-Mental State Examination), GDSCALE (Geriatric Depression Scale), Global CDR (Clinical Dementia Rating), FAQ-Score (Functional Activities Questionnaire), and NPIQ-Score (Neuropsychiatric Inventory Questionnaire). For each subject, 10 slices were extracted from their 3D MRI scans, totaling 2000 images. A summary of the dataset distribution for the experiments is provided in Table 1. To ensure a robust evaluation and prevent data leakage, data splitting was conducted at the patient level. All slices from a single subject were assigned exclusively to one set (training, validation, or testing), preventing any slices from the same subject from appearing in multiple sets. Specifically, 20% of the subjects were set aside as a held-out test set, which was not used during training or model selection. The remaining 80% of subjects were employed for subject-level 5-fold cross-validation, with each fold maintaining strict separation between training and validation sets. Model performance during cross-validation was reported as the mean ± standard deviation across folds. The configuration with the best average performance was then retrained on the entire training–validation set and finally evaluated on the held-out test set to provide an unbiased estimate of the model’s performance.

Additionally, external validation was conducted using the OASIS-3 dataset, a large-scale, publicly accessible neuroimaging resource featuring longitudinal MRI scans, cognitive assessments, and clinical data across the cognitive spectrum. The same preprocessing steps were applied to maintain consistency, allowing for a fair evaluation of the model’s generalizability beyond the ADNI1 cohort. The OASIS-3 subset comprised 704 NC, 19 MCI, and 198 AD images (total 921 for external validation). In addition to imaging, it offers rich metadata, including demographics, diagnoses, and longitudinal clinical measures and supporting comprehensive subject characterization.

### 4.2. Feature Selection

Selecting the most important features is crucial for enhancing model performance, reducing dimensionality, and improving interpretability. The original feature set included Weight, Age, APOE-A1, APOE-A2, MMSE, GDSCALE, Global-CDR, FAQ-Score, and NPIQ-Score, though not all may significantly contribute to the performance. To identify the most relevant features, we first extracted metadata features from the dataset and normalized them using StandardScaler to ensure comparability across different scales. We specifically chose Random Forest, XGBoost, LightGBM, Extra Trees, and AdaBoost for their diverse strengths in handling structured data and effectively capturing feature importance. Next, we trained five ensemble models on the processed data and extracted feature importance scores from each model. To ensure robustness, we applied a majority voting mechanism, where features received weighted points based on their rankings across all models, with higher-ranked features accumulating more points.

**Figure 2:**
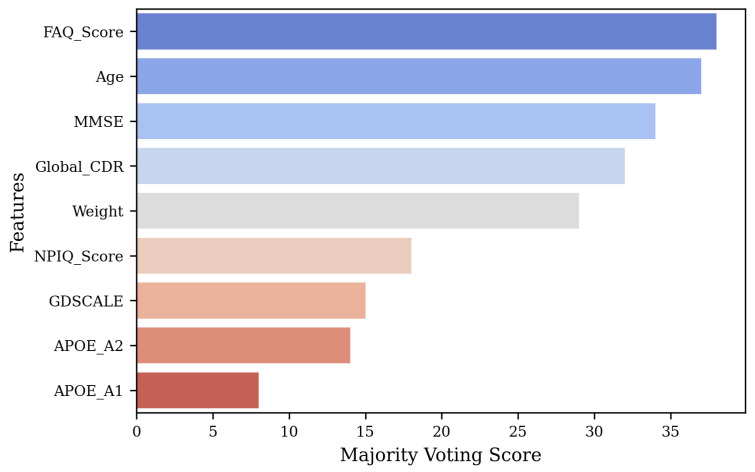
Majority voting scores for feature importance across different ensemble models.

Let $r_{m,f}$ denote the rank of feature *f* assigned by ensemble model *m*. We compute a weighted vote $v_{f}=\sum_{m}w_{m}\left(\frac{1}{r_{m,f}}\right)$, where $w_{m}$ is the weight of model *m* (equal weights in our case). The scores are normalized as $s_{f}=\frac{v_{f}}{\sum_{f^{\prime }}v_{f^{\prime }}}$ and the features with the top k=5 scores are selected. The total votes for each feature were then normalized to compute confidence scores, representing the proportion of votes received relative to the total. Finally, the features were ranked based on their majority voting scores, with the top five retained features being FAQ-Score, Age, MMSE, Global-CDR, and Weight, which demonstrated the highest importance across all models. Figure 2 shows the feature selection results.

### 4.3. Implementation

We implemented the NeuroNet-AD model to evaluate the performance across different experimental settings. The model’s training process was performed using PyTorch. We employed the cross-entropy loss function, which is suitable for multiclass classification tasks. This loss function measures the performance of the model by comparing the predicted class probabilities with the actual class labels. The model parameters were optimized using the Adam optimizer. The learning rate was set to 0.001, providing a balance between convergence speed and stability. To enhance generalization and reduce overfitting, we employed several regularization techniques. Specifically, dropout layers with a rate of 0.5 were added to the fully connected layers, and weight decay regularization (set to $10^{-5}$) was included in the Adam optimizer to penalize overly complex models. Additionally, data augmentation techniques were applied to the MRI slices, including random horizontal flipping, small-angle rotations (±10°), and slight intensity scaling, thereby increasing the effective diversity of the training data.

To mitigate the risk of overfitting, we incorporated an early stopping mechanism with a patience of 20 epochs, meaning the training process was terminated if the validation accuracy did not improve for 20 consecutive epochs. We monitored the validation accuracy, and the model achieving the highest validation accuracy was saved and considered the best-performing model for that specific experimental setup. Given the relatively small dataset size, all splits were performed at the patient level to avoid data leakage, and model evaluation followed subject-level 5-fold cross-validation with an independent held-out test set.

To strictly enforce subject-level independence and prevent data leakage between slices, we grouped all MRI slices by unique subject identifiers before partitioning. This ensured that slices from the same subject were never split across training, validation, or test sets. During 5-fold cross-validation, we performed stratified sampling at the subject level to maintain approximately balanced distributions of NC, MCI, and AD subjects across all folds. In each fold, one set of subjects was reserved exclusively for validation, while the remaining were used for training, ensuring no overlap between partitions. After cross-validation, we retrained the best-performing configuration on the full training–validation set and then tested it on the held-out test set, which included 20% of subjects unseen during both training and model selection. The complete implementation of the splitting pipeline, including the subject-ID–based partitioning code, is publicly available in our GitHub repository (https://github.com/Rahman-Motiur/NeuroNet-AD) to ensure full reproducibility.

## 5. Results and Discussion

This research study focuses on classifying multiclass AD (NC vs. MCI vs. AD) using MRI images and their corresponding textual metadata. We evaluated the performance of NeuroNet-AD and multiple baseline models over the ADNI1 dataset in terms of accuracy, precision, recall, and F1-score, followed by validation on an independent held-out test set and the external OASIS-3 dataset. In addition, ablation studies were conducted to quantify the contribution of each NeuroNet-AD component.

### 5.1. Comparison with State-of-the-Art Models

Table 2 and Table 3 provide a comparative overview of NeuroNet-AD against several state-of-the-art (SOTA) approaches across multiple datasets and modalities. For multiclass classification (NC vs. MCI vs. AD), NeuroNet-AD achieved the highest accuracy of 98.68% on the ADNI dataset, surpassing recent multimodal models such as MADDi [42] (96.88%) and vision–language frameworks like MedBLIP [54] (78.7–85.3%). In the binary classification setting, NeuroNet-AD also outperformed traditional and hybrid approaches, including the hybrid model of [52], which reported 98.4% accuracy on NC vs. AD, a comparatively less-complex task. These results demonstrate that NeuroNet-AD not only delivers higher accuracy but also tackles the more challenging multiclass classification problem, thereby establishing a new benchmark in AD diagnosis.

### 5.2. Cross-Validation and Held-Out Test Performance on ADNI1

To ensure robust evaluation and minimize data leakage, subject-level 5-fold cross-validation was performed on 80% of the ADNI1 dataset using NeuroNet-AD along with the popular CNN and vision transformer (ViT) models. As summarized in Table 4, NeuroNet-AD achieved an average accuracy of 98.20±0.50% across folds, consistently outperforming baseline CNNs (ResNet18, VGG16, MobileNet, and EfficientNet) and the transformer model (ViT) by more than 5%. In addition, NeuroNet-AD demonstrated the lowest variance across folds, suggesting both stable learning and strong generalization potential. These results confirm that the proposed multimodal architecture effectively leverages both imaging and clinical metadata to enhance predictive performance.

To confirm that the observed improvements were not caused by random variation, we performed paired *t*-tests comparing NeuroNet-AD to each baseline model across five cross-validation folds. Table 5 shows the mean accuracy with standard deviation, 95% confidence intervals (CI), and *p*-values. In all cases, NeuroNet-AD’s performance improvements over CNN and transformer baselines were statistically significant (p<0.05). These findings provide strong statistical evidence that NeuroNet-AD’s improvements are consistent rather than due to sampling variability.

After retraining on the full 80% training–validation pool, NeuroNet-AD was evaluated on the independent held-out 20% test set comprising subjects never seen during training. Table 6 presents the class-wise and overall results. NeuroNet-AD achieved an overall accuracy of 98.68% with balanced precision, recall, and F1-scores across NC, MCI, and AD classes. Importantly, the model showed no significant performance drop compared to cross-validation, indicating that the network generalizes well to unseen patients. The near-perfect recall for AD (99.40%) is particularly encouraging as it reflects high sensitivity in detecting patients with Alzheimer’s disease, a critical aspect for clinical utility.

**Figure 3:**
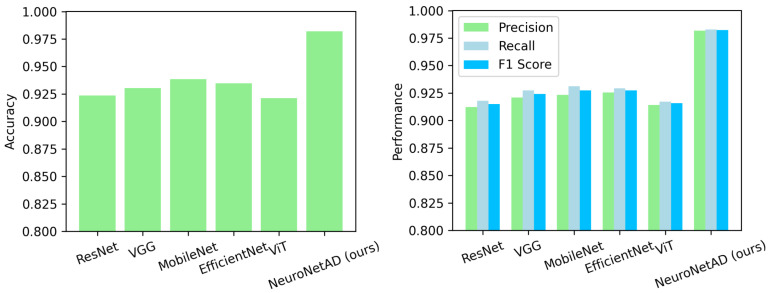
Performance comparison of different models based on four key evaluation metrics: accuracy, precision, recall, and F1-score.

Figure 3 compares NeuroNet-AD with several baseline models across four evaluation metrics: accuracy, precision, recall, and F1-score. Across all metrics, NeuroNet-AD consistently demonstrates superior performance with notable improvements over competing methods.

**Figure 4:**
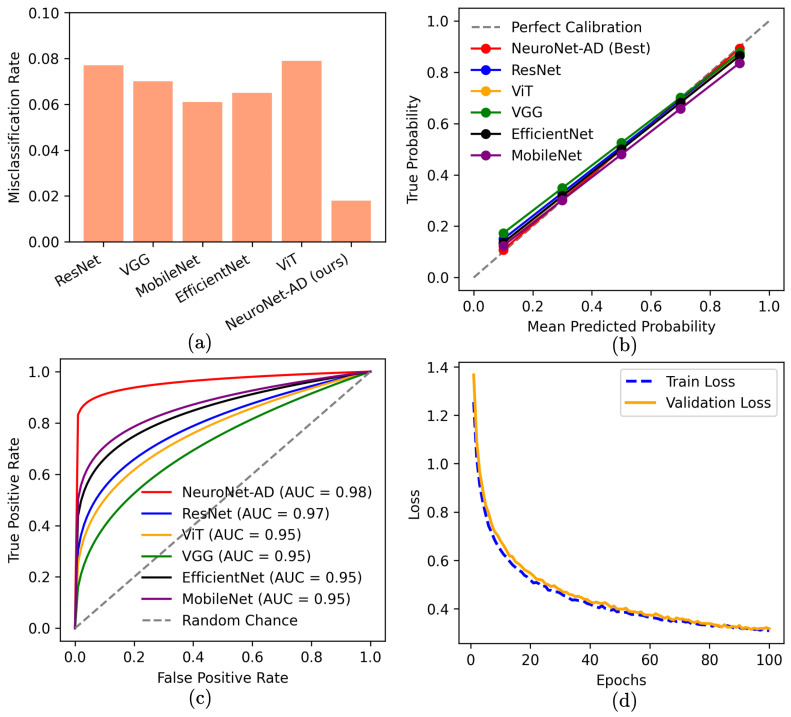
Performance of NeuroNet-AD vs. baselines: (**a**) misclassification rates (lowest for NeuroNet-AD), (**b**) calibration curves (best alignment), (**c**) Receiver Operating Characteristics (ROC) curves with Area Under Curve (AUC), and (**d**) training/validation loss over epochs (smooth convergence).

Figure 4 provides deeper insights into model behavior: (a) NeuroNet-AD exhibits the lowest misclassification rates. (b) The calibration curve shows that NeuroNet-AD’s probability estimates align most closely with the ideal diagonal, indicating that its predicted confidence levels faithfully reflect true outcome frequencies. Other baseline models exhibit slight deviations, suggesting mild over- or under-confidence compared to NeuroNet-AD. (c) Receiver Operating Characteristics (ROC) show NeuroNet-AD achieving the highest Area Under Curve (AUC) (0.98) among all models. (d) The training and validation loss curves illustrate smooth convergence and strong generalization with minimal overfitting.

### 5.3. External Validation on OASIS-3

To further assess generalization across datasets and acquisition protocols, NeuroNet-AD was evaluated on the OASIS-3 dataset using identical preprocessing. Despite variations in scanner hardware, demographics, and disease distribution, the model maintained strong performance in the multiclass setting (NC vs. MCI vs. AD), achieving 94.10% accuracy with balanced precision, recall, and F1-scores. Furthermore, in the binary classification task (NC vs. AD), NeuroNet-AD reached 98.67% accuracy with consistently high precision, recall, and F1-scores (Table 7). These results highlight NeuroNet-AD’s robustness beyond the ADNI cohort and confirm its adaptability to real-world clinical variability across both binary and multiclass diagnostic scenarios.

### 5.4. Ablation Studies

We conducted ablation studies to evaluate the contribution of each NeuroNet-AD component. Table 8 presents the incremental gains achieved by adding CBAM, MGCA, feature selection (FS), and text encoder (TE) modules. The baseline ResNet18 model achieved an accuracy of 93.13%. Adding CBAM improved accuracy to 94.97%, confirming the benefit of channel–spatial attention in highlighting discriminative brain regions. Incorporating MGCA for cross-modal fusion further boosted accuracy to 96.67%, while feature selection of the most relevant metadata features raised accuracy to 97.89%. Finally, integrating a BERT-based text encoder improved performance to 98.68%, demonstrating the importance of semantic text embeddings in enhancing multimodal representation.

A complementary component-removal ablation study (Table 9) confirmed these findings. In a leave-one-component-out setting, where one element is removed while all other components and the training protocol remain fixed, omitting CBAM, MGCA, feature selection, or the text encoder resulted in absolute performance drops of 3% to 6%. The largest reductions in performance were observed when either MGCA (cross-attention fusion) or CBAM was removed. The image-only baseline, which used ResNet-18 without metadata fusion, performed the worst with an accuracy of 91.20%. These results indicate that each component contributes significantly on its own, and their synergistic integration is crucial for NeuroNet-AD’s superior accuracy and robustness.

To experimentally justify our decision to divide the embedding into four parts in the MGCA module, we performed an ablation study by changing the number of attention heads. As shown in Table 10, increasing the number of attention heads from one to four consistently improves model performance, with accuracy increasing from 96.42% (single-head) to 98.68% (four heads). The improvements in precision, recall, and F1-score show that moderate multi-head partitioning helps MGCA capture more complex cross-modal interactions between MRI and clinical metadata. Meanwhile, the computational overhead in Floating-Point Operations (FLOPs) only rises slightly (3.12G to 3.78G). However, further increasing to eight heads raises FLOPs (4.42G) without additional performance gains; in fact, accuracy slightly drops to 97.89%. This indicates that over-fragmenting the embedding space decreases per-head capacity while adding more projection costs. Overall, these findings confirm that using four heads achieves the best balance between accuracy and computational efficiency in the MGCA module.

**Figure 5:**
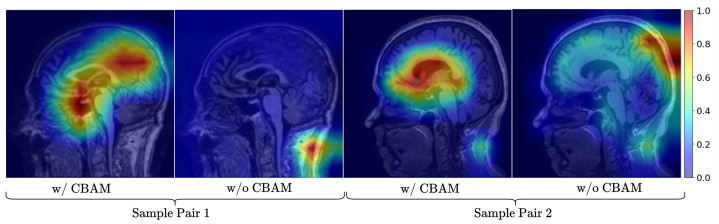
The figure demonstrates that the model with CBAM focuses more precisely on relevant features compared to the model without CBAM. We computed Class Activation Maps (CAMs) using the feature maps from the conv3 layer of the final bottleneck block in model.layer4 (i.e., model.layer4[-1].conv3) of the ResNet architecture. Each CAM pair (left: without CBAM; right: with CBAM) is aligned for the same input MRI slice to visually demonstrate CBAM’s effect. The resulting visualizations highlight that the model focuses on the relevant spatial regions to make class predictions.

We further provide visualizations to illustrate the contribution of CBAM to feature selection. The Class Activation Map (CAM) results (Figure 5) for two different sample pairs clearly demonstrate that CBAM guides the model to focus on clinically relevant brain regions, thereby enhancing interpretability.

These findings, collectively, confirm that the proposed NeuroNet-AD framework not only improves predictive performance but also strengthens robustness and interpretability for clinical AD diagnosis.

## 6. Conclusions

In this study, we proposed NeuroNet-AD, a novel multimodal deep learning framework designed to improve the diagnosis of AD, including its early stage, known as MCI. NeuroNet-AD effectively integrates structural MRI images with clinical text-based metadata, utilizing complementary information from both modalities to enhance diagnostic accuracy. Incorporating the Convolutional Block Attention Module (CBAM) within the ResNet-18 backbone significantly improved image feature extraction by emphasizing critical spatial and channel-wise information. The Meta-Guided Cross-Attention (MGCA) module also facilitated robust cross-modal feature alignment, enabling effective fusion of neuroimaging and textual data. Our ensemble-based feature selection strategy enhanced model performance by identifying the most discriminative features from the clinical metadata, reducing overfitting, and improving generalization. We evaluated NeuroNet-AD on the ADNI1 dataset using subject-level 5-fold cross-validation and a held-out test set to ensure robustness. NeuroNet-AD achieved 98.68% accuracy in multiclass classification tasks and 99.13% accuracy in the binary setting on the ADNI dataset, outperforming state-of-the-art models. External validation on the OASIS-3 dataset further confirmed the model’s generalization ability, achieving 94.10% accuracy in the multiclass setting and 98.67% accuracy in the binary setting, despite demographic and acquisition variability. The effectiveness of CBAM, MGCA, and advanced feature engineering in improving diagnostic performance was further validated through comprehensive ablation studies. A limitation of this work is that variations in test-set definitions across studies may hinder direct performance comparisons, even though we employed a strict subject-level split with an independent held-out test set. Another limitation is that the model is currently restricted to MRI and limited clinical metadata, which may reduce generalizability and interpretability across diverse patient populations. In future studies, we aim to explore the integration of additional modalities such as genetic data, longitudinal patient records, and other neuroimaging techniques to further enhance the model’s diagnostic capabilities and interpretability in real-world clinical settings.

## Figures and Tables

**Table 1:** Summary of ADNI1 dataset split (200 subjects, 2000 slices). Data splitting was performed at the patient level to prevent leakage. The dataset was divided into a held-out test set (20% of subjects) and a cross-validation pool (80%). Within the cross-validation (CV) pool, 5-fold cross-validation was applied, with 4 folds for training and 1 fold for validation in each round.

	Held-Out Test (20%)		CV Pool (80%)		Per Fold (Within CV Pool)	
Class	Subjects	Slices	Subjects	Slices	Train	Validation
NC	12	120	48	480	38–39	9–10
MCI	14	140	56	560	44–45	11–12
AD	14	140	56	560	44–45	11–12
Total	40	400	160	1600	128	32

**Table 2:** Performance (accuracy) comparison of NeuroNet-AD with SOTA models for AD diagnosis (binary classification).

Model	Dataset (Size|Test)	Modality	Performance (%)
SVM + Bayes [59]	Bordeaux (37|-)	MRI + CSF	85.0
SVM + Bayes [59]	ADNI (218|-)	MRI + CSF	87.0
SVM [60]	ADRC (380|100)	sMRI + Demographics + APOE	88.5
GNN + VLM [55]	ADReSSo (548|-)	Imaging + Text	88.73
SVM [60]	ADRC (380|100)	sMRI + APOE	89.3
LLM + CNN + Transformers [56]	ADNI (618|103)	Imaging + Text	94.71 (EMCI vs. LMCI)
LLM + CNN + Transformers [56]	ADNI (618|103)	Imaging + Text	96.36 (NC vs. AD)
Hybrid Model [52]	ADNI (–)	MRI + Clinical Features	98.4
**NeuroNet-AD (Proposed)**	ADNI (2000|400)	MRI + Clinical + Text	**99.13 (NC vs. AD)**

**Table 3:** Performance (accuracy) comparison of NeuroNet-AD with SOTA models for AD diagnosis (multiclass classification).

Model	Dataset (Size|Test)	Modality	Performance (%)
MedBLIP [54]	ADNI (10,387|600)	3D Images + Text	78.7
MedBLIP [54]	NACC (15,354|600)	3D Images + Text	83.3
MedBLIP [54]	OASIS (3,020|400)	3D Images + Text	85.3
MADDi [42]	ADNI (717|72)	Imaging + Genetic + Clinical	96.88
**NeuroNet-AD (Proposed)**	ADNI (2000|400)	MRI + Clinical + Text	**98.68 (NC vs. MCI vs. AD)**

**Table 4:** Mean ± standard deviation of performance metrics across 5-fold cross-validation on the training–validation set. NeuroNet-AD consistently outperformed baseline models, demonstrating stable learning behavior.

Model	Accuracy (%)	Precision (%)	Recall (%)	F1-Score (%)
NeuroNet-AD	98.20±0.50	98.18±0.62	98.28±0.58	98.23±0.55
ResNet18	92.35±1.12	91.22±1.35	91.80±1.24	91.51±1.29
VGG16	93.04±1.05	92.10±1.12	92.74±1.09	92.42±1.10
MobileNet	93.85±0.98	92.34±1.08	93.12±1.02	92.73±1.05
EfficientNet	93.48±1.01	92.56±1.15	92.93±1.07	92.74±1.12
ViT	92.12±1.20	91.43±1.34	91.72±1.28	91.57±1.31

**Table 5:** Statistical significance testing of NeuroNet-AD in comparison with baseline models on the ADNI1 dataset using subject-level 5-fold cross-validation. Values are reported as mean accuracy ± standard deviation across folds, along with paired *t*-test *p*-values against NeuroNet-AD.

Model	Accuracy (%)	95% CI (%)	*p*-Value vs. NeuroNet-AD
ResNet-18	92.35 ± 1.12	[91.20, 93.50]	<0.02
VGG16	93.04 ± 1.05	[91.90, 94.18]	<0.03
MobileNet	93.85 ± 0.98	[92.78, 94.92]	<0.01
EfficientNet	93.48 ± 1.01	[92.35, 94.61]	<0.03
ViT	92.12 ± 1.20	[90.82, 93.42]	<0.02
**NeuroNet-AD**	**98.20 ± 0.50**	[97.65, 98.75]	–

**Table 6:** Performance of NeuroNet-AD on the held-out 20% test set (subjects never seen during training or validation).

Class	Accuracy (%)	Precision (%)	Recall (%)	F1-Score (%)
NC	98.75	99.10	98.40	98.75
MCI	98.50	98.30	98.70	98.50
AD	98.80	98.20	99.40	98.80
Overall	98.68	98.53	98.83	98.66

**Table 7:** External validation of NeuroNet-AD on the OASIS-3 dataset. Despite differences in scanner hardware, demographics, and disease distribution, the model maintained strong performance in both multiclass and binary settings.

Model	Accuracy (%)	Precision (%)	Recall (%)	F1-Score (%)
NeuroNet-AD (Multiclass)	94.10	94.05	94.20	94.12
NeuroNet-AD (Binary)	98.67	98.45	98.72	98.36

**Table 8:** Ablation studies of different components of NeuroNet-AD.

Base Model	CBAM	MGCA	Meta Data	Feature Selection (FS)	Text Encoder (TE)	Accuracy
✓						93.13
✓	✓					94.97
✓	✓	✓	✓			96.67
✓	✓	✓	✓	✓		97.89
✓	✓	✓	✓	✓	✓	98.68

**Table 9:** Component-wise ablation study of NeuroNet-AD on the ADNI1 held-out test set. Each row reports performance when a component is removed from the full model.

Variant	Accuracy (%)	Precision (%)	Recall (%)	F1-Score (%)
Full (CBAM + MGCA + FS + TE)	98.68	98.53	98.83	98.66
CBAM	93.43	93.21	92.93	93.09
MGCA	92.85	92.73	92.25	92.54
Feature Selection (FS)	94.28	94.04	93.87	93.81
Text Encoder (TE)	93.54	93.38	93.07	93.18
No Fusion (image-only ResNet-18)	91.20	91.08	90.81	90.97

**Table 10:** Effect of the number of attention heads in the MGCA module on classification performance and computational complexity (ADNI1 held-out test set). FLOPs are reported in billions (i.e., $10^{9}$ or Giga (G)).

Number of Heads	FLOPs (G)	Accuracy (%)	Precision (%)	Recall (%)	F1-Score (%)
1 (Single-Head)	3.12	96.42	96.20	96.35	96.27
2	3.35	97.25	97.18	97.22	97.20
**4 (Proposed)**	**3.78**	**98.68**	**98.53**	**98.83**	**98.66**
8	4.42	97.89	97.60	97.78	97.69

## Data Availability

The data analyzed in this study was mainly obtained from the Alzheimer’s Disease Neuroimaging Initiative (ADNI) dataset, available from https://adni.loni.usc.edu/ (accessed on 12 October 2025). The external validation data analyzed in this study was obtained from the Open Access Series of Imaging Studies (OASIS) dataset, available from https://sites.wustl.edu/oasisbrains/ (accessed on 12 October 2025). The full code of NeuroNet-AD and data of this study is available on GitHub: https://github.com/Rahman-Motiur/NeuroNet-AD (accessed on 12 October 2025).
